# Oseltamivir Treatment for Children with Influenza-Like Illness in China: A Cost-Effectiveness Analysis

**DOI:** 10.1371/journal.pone.0153664

**Published:** 2016-04-15

**Authors:** Kunling Shen, Tengbin Xiong, Seng Chuen Tan, Jiuhong Wu

**Affiliations:** 1 Department of Respiratory Care, Beijing Children’s Hospital, Beijing, China; 2 Health Economics & Outcomes Research, IMS Health China, Shanghai, China; 3 Health Economics & Outcomes Research, IMS Health Asia Pacific, Singapore, Singapore; 4 Department of Pharmacy, The 306th Hospital of PLA, Beijing, China; University of British Columbia, CANADA

## Abstract

**Background:**

Influenza is a common viral respiratory infection that causes epidemics and pandemics in the human population. Oseltamivir is a neuraminidase inhibitor—a new class of antiviral therapy for influenza. Although its efficacy and safety have been established, there is uncertainty regarding whether influenza-like illness (ILI) in children is best managed by oseltamivir at the onset of illness, and its cost-effectiveness in children has not been studied in China.

**Objective:**

To evaluate the cost-effectiveness of post rapid influenza diagnostic test (RIDT) treatment with oseltamivir and empiric treatment with oseltamivir comparing with no antiviral therapy against influenza for children with ILI.

**Methods:**

We developed a decision-analytic model based on previously published evidence to simulate and evaluate 1-year potential clinical and economic outcomes associated with three managing strategies for children presenting with symptoms of influenza. Model inputs were derived from literature and expert opinion of clinical practice and research in China. Outcome measures included costs and quality-adjusted life year (QALY). All the interventions were compared with incremental cost-effectiveness ratios (ICER).

**Results:**

In base case analysis, empiric treatment with oseltamivir consistently produced the greatest gains in QALY. When compared with no antiviral therapy, the empiric treatment with oseltamivir strategy is very cost effective with an ICER of RMB 4,438. When compared with the post RIDT treatment with oseltamivir, the empiric treatment with oseltamivir strategy is dominant. Probabilistic sensitivity analysis projected that there is a 100% probability that empiric oseltamivir treatment would be considered as a very cost-effective strategy compared to the no antiviral therapy, according to the WHO recommendations for cost-effectiveness thresholds. The same was concluded with 99% probability for empiric oseltamivir treatment being a very cost-effective strategy compared to the post RIDT treatment with oseltamivir.

**Conclusion:**

In the Chinese setting of current health system, our modelling based simulation analysis suggests that empiric treatment with oseltamivir to be a cost-saving and very cost-effective strategy in managing children with ILI.

## Introduction

The influenza virus, consist of the influenza A and the influenza B subgroups, known as a circulating pathogen in the human population since the 16th century, is notable for its unique ability to cause recurrent epidemics and global pandemics [1]. Symptoms of influenza include fever, cough, headache, sore throat, nasal congestion, fatigue, and joint and muscle aches.

In China, strategies for managing the symptoms of uncomplicated seasonal influenza illness in children, include over-the-counter (OTC) drugs from the pharmacy services; or having a rapid influenza diagnostic test (RIDT) and obtaining an antiviral therapy when result is positive; or getting the empiric antiviral therapy directly.

Antiviral therapy could reduce the duration and severity of disease, and limit further spread of an outbreak [2]. With the development of neuraminidase inhibitors, oseltamivir has been approved for treatment of seasonal influenza in children aged ≥ 1 year and licensed for therapeutic and prophylactic use, oseltamivir is active against both influenza A and B [3].

Early oseltamivir treatment can reduce signs and symptoms, shorten the illness duration of influenza, improve clinical outcomes and decrease the development of serious complications such as otitis media in young children [4]. According to the current guidelines of influenza diagnosis and treatment in China, oseltamivir is used as the basic first-line antiviral therapy for influenza patients, and to be effective, antiviral therapy should be started within 48 hours of symptom onset.

Up to now, several economic evaluations of oseltamivir have been conducted from different perspectives in multiple countries including the US, UK, Japan and Denmark [5–8]. However, no comprehensive cost-effectiveness analysis has been conducted on the treatment of children with influenza-like-illness (ILI) in China. The objective of this analysis was to evaluate the cost effectiveness of the antiviral therapies by oseltamivir treatment, which delivered in two ways, i.e. post RIDT treatment with oseltamivir and empiric treatment with oseltamivir, compared to no antiviral therapy in children with symptoms of influenza, and to estimate the incremental cost-effectiveness ratios (ICER) of the above interventions in comparisons with each other.

## Methods

### Model Design

**Fig 1:**
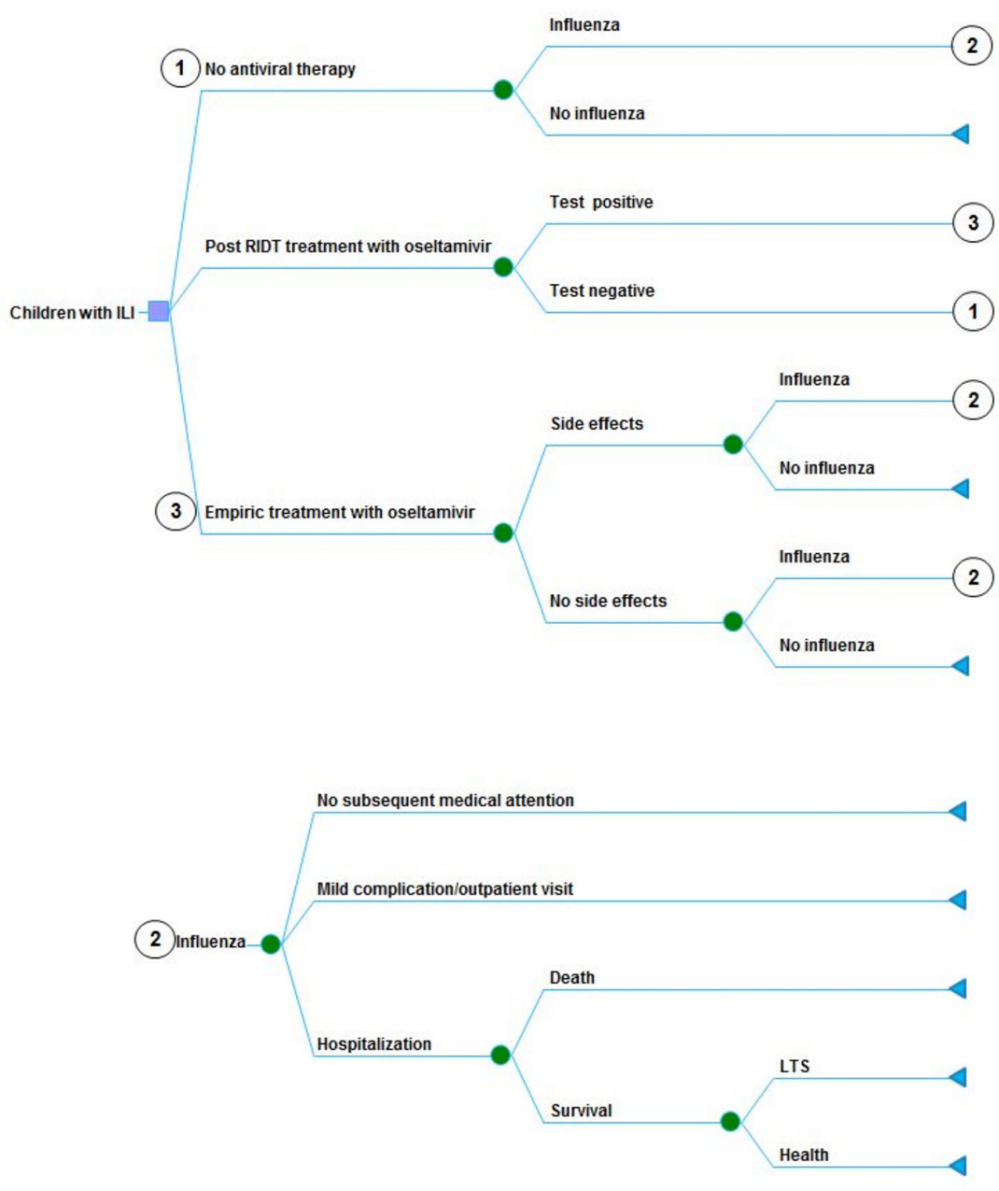
Simplified diagram of decision analytic model.

A decision-analytic model based on previously published evidence [5] was designed to simulate and evaluate 1-year and life-time potential clinical and economic outcomes associated with three management strategies for children with ILI: no antiviral therapy; RIDT for influenza, followed by treatment with oseltamivir when results were positive; and empiric treatment with oseltamivir for children with symptoms of influenza (Fig 1). The RIDT used in the model is the colloidal gold immunochromatographic assay (GICA), which is widely used in China for rapid detection of influenza.

In the model, case inclusion criteria were children aged 18 years or below with ILI, had symptoms and signs compatible with influenza (e.g. fever, cough). The children with ILI might or might not be infected with influenza. In the “no antiviral therapy” arm, children with ILI would only receive the OTC for symptoms without any antiviral therapy. In the “post RIDT treatment with oseltamivir” arm, children would receive oseltamivir when testing results were positive. In the “empiric treatment with oseltamivir” arm, all children with ILI would receive a full course of oseltamivir.

Although the oseltamivir-resistant influenza has been reported in adults during the influenza season [9, 10], in the analysis we did not consider the assumptions of oseltamivir resistance and influenza vaccination, as the oseltamivir-resistant influenza were rarely found in children in China [11], and the vaccination rate and willingness of vaccination in children were low in China [12]. The model is developed from the perspective of local health system and setting in China. The costs included in this analysis were drug costs, cost of complication treatment, cost of long term sequelae (LTS) and cost of RIDT.

### Epidemiology

Chinese epidemiological and resource use data for influenza were obtained from literature search and review of local studies in China. Published overseas data will be considered and used as a proxy in the absence of local estimates. For an unvaccinated child coming to a physician’s office with uncomplicated ILI, we used a probability of seasonal influenza virus infection as 39.3% (ranging from 23.7% to 54.9%), on the basis of published data of medically attended [13].

The model included influenza-related complications such as otitis media, pneumonia, hospitalization (with and without long-term sequelae), and death (Table 1 and S1 Table). In the base case analysis, we considered that oseltamivir treatment would shorten the duration of uncomplicated influenza symptoms [5] and reduce the likelihood of mortality [14]. We also assumed that oseltamivir treatment would reduce the incidence of influenza-related otitis media and non-hospitalized pneumonia by 40% [4], but would have no effect on the incidence of the other influenza-related outcomes included in the model including hospitalization and long-term sequelae [5, 14].

**Table 1:** Model parameters for base case analysis.

Variable	Base case	Reference
Probability that a child with ILI has influenza	39.30%	[13]
Probability of otitis media for a child with medically attended influenza illness	18.10%	[17]
Probability of non-hospitalized pneumonia with medically attended influenza illness	15.12%	[18]
Probability of other uncomplicated outpatient influenza-related complications	17.00%	[13]
Probability of hospitalizations for respiratory condition due to influenza	1.09%	[5]
Probability of long-term sequelae after influenza-related hospitalization	1.00%	[5]
Probability of dying during influenza-related hospitalization	0.20%	[19]
Probability of medically attended oseltamivir-related adverse events		
Gastrointestinal symptoms	7.45%	[20–24]
Neuropsychiatric symptoms	0.07%	[16]
Costs (2015 RMB)		
Over-the Countcer medications	RMB21.34	[25]
Oseltamivir	RMB69.00	[26]
Physician visit for uncomplicated influenza	RMB429.08	[13]
Physician visit for otitis media	RMB279.70	[27, 28]
Physician visit for non-hospitalized pneumonia	RMB938.38	[29–33]
Hospitalization for influenza-related complication	RMB3,934.00	[34]
Physician visit for gastrointestinal symptoms	RMB60.00	[16]
Episode cost of neuropsychiatric symptoms	RMB0.00	[16]
RIDT	RMB42.84	[13]
1-year cost of long term sequelae	RMB12,622.02	[35]
Characteristics of RIDT		
Sentivity	26.67%	[15]
Specificity	78.95%	[15]
Oseltamivir treatment efficacy		
Reduction in the duration of uncomplicated influenza symptoms	26.00%	[5]
Reduction in risk of otitis media	40.00%	[4]
Reduction in risk of pneumonia	40.00%	[4]
Odds ratio in mortality	0.82	[14]
Disutility values		
Episode of influenza	0.005	[5]
Episode of otitis media	0.042	[5]
Episode of pneumonia (or other non-hospitalized complication)	0.046	[5]
Hospitalization for pneumonia due to influenza	0.076	[5]
Gastrointestinal adverse effect	0.0013	[5]
Neuropsychiatric antiviral-related adverse event	0.0029	[5]
Utility of long term sequelae	0.56	[36]

The sensitivity (26.67%) and specificity (78.95%) of RIDTs were estimated from published studies and expert opinion [15]. Gastrointestinal symptoms and neuropsychiatric events were included as oseltamivir-related adverse events in the model. According to the published studies the oseltamivir-related adverse events were conservatively assumed [16], if oseltamivir-related adverse events result in the discontinuation of therapy, the full cost of treatment was incurred but there will be no treatment benefit.

### Costs and Outcome Measures

The medical costs for the healthcare resources used in each strategy include the costs of outpatient visits, RIDTs, medications, emergency department visits, and hospitalizations.

For the children with ILI to pick “no antiviral therapy” (the branch ① in Fig 1), the cost of OTC medications will occur, and no further cost for the children *without* influenza; however, for those children *with* influenza (the branch ② in Fig 1), the further costs *may* occur including physician visit for uncomplicated influenza, or otitis media, or non-hospitalized pneumonia, or hospitalization for serious complications or costs for LTS (Table 1).

For the children with ILI to seek the empiric antiviral therapy directly (the branch ③ in Fig 1), the acquisition cost of oseltamivir will occur, and further costs *may* happen for the medically attended oseltamivir-related side effects, including the costs of physician visit for gastrointestinal or neuropsychiatric symptoms (Table 1). The cost of RIDT will occur for those children with ILI having RIDT and obtaining an antiviral therapy when the result is positive.

Disutility values were used to account for the morbidity associated with uncomplicated influenza, influenza related complications, and oseltamivir related adverse events [5]. These disutility values were obtained from primary data and published literature.

### Cost-effectiveness Analyses

Two competing strategies were compared in terms ICERs, which are calculated by dividing the difference in costs by the difference in QALYs over a time horizon of 1 year. Several one-way sensitivity analyses were performed to rigorously test the cost-effectiveness results. The parameters included in one-way sensitivity analyses are listed in S1 Table, including the sensitivity analyses performed. Results were displayed in a tornado diagram to demonstrate the effect of the variable on the QALY and ICER.

Additionally, a probabilistic sensitivity analysis was performed to test the robustness of the ICER result by simultaneously varying the values of all input parameters which were sampled from assigned distributions to obtain 1,000 mean incremental values that were plotted on a cost-effectiveness plane. Based on WHO recommendations for cost-effectiveness thresholds [37], we describe an intervention as very cost-effective if the estimated ICER is less than per capita GDP in China (RMB46,629 in 2014) and cost-effective if it is less than three times per capita GDP in China (RMB139,887) [38].

## Results

### Base Case Analysis

For a cohort of children not vaccinated against seasonal influenza coming to the physician’s office with ILI, empiric treatment with oseltamivir would cost RMB 14 more than the no antiviral therapy for each child, and produced the greatest number of expected QALYs among all groups. This treatment strategy was estimated to cost additional RMB4,438 per QALY gained, compared with no antiviral therapy. The empiric treatment with oseltamivir strategy is dominant (generating more QALYs with less estimated cost) compared with post RIDT treatment with oseltamivir. The testing strategy (RIDT) was projected to cost RMB32,810 per QALY gained, compared with the no oseltamivir treatment strategy (Table 2).

**Table 2:** Incremental cost-effectiveness ratios for influenza management.

Strategies	No antiviral therapy (A)	Post RIDT treatment with oseltamivir (B)	Empiric treatment with Oseltamivir (C)	
**Costs**	RMB143	RMB172	RMB157	
Medication + test cost	RMB21	RMB61	RMB69	
Treatment cost	RMB122	RMB111	RMB88	
**QALY**	0.9926	0.9935	0.9959	
**Incremental Analysis**	-	B vs. A	C vs. B	C vs. A
Δ Cost		RMB28.53	- RMB14.11	RMB14.41
Δ QALY		0.0009	0.0024	0.0032
**ICER**		RMB32,810	Dominance	RMB4,438

### One-way Sensitivity Analysis

**Fig 2:**
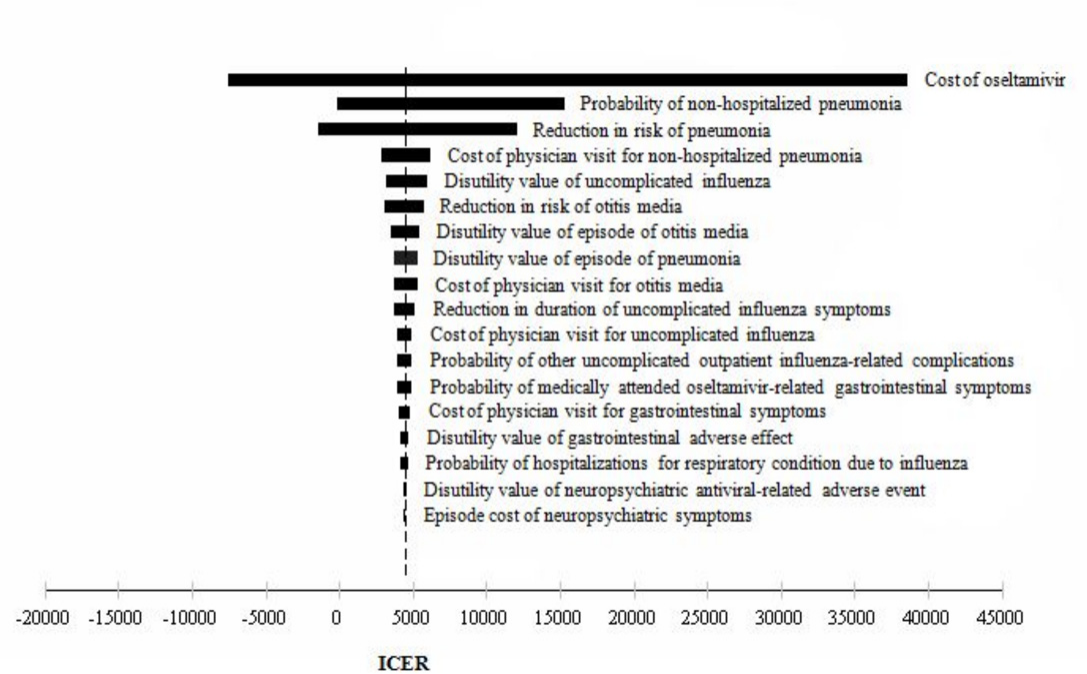
One-way sensitivity analyses tornado diagram: Empiric treatment with oseltamivir vs. No antiviral therapy.

**Fig 3:**
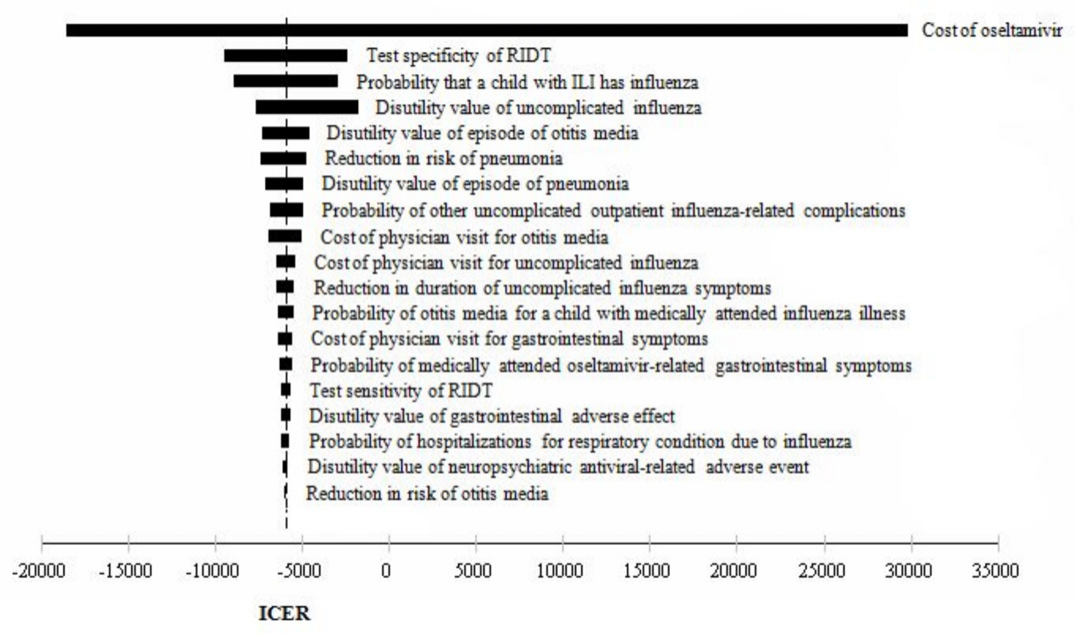
One-way sensitivity analyses tornado diagram: Empiric treatment with oseltamivir vs. post RIDT treatment with oseltamivir.

One-way sensitivity analysis results are presented in Fig 2 and Fig 3. In comparison between empiric treatment with oseltamivir and no antiviral therapy, the ICER was sensitive to changes in inputs such as cost of oseltamivir, probability of non-hospitalized pneumonia, and reduction in risk of pneumonia by oseltamivir.

Similarly, different values in model inputs such as cost of oseltamivir, specificity of RIDT, and the probability that a child with ILI has influenza were shown to cause relatively large variation in ICER of comparison between empiric treatment with oseltamivir and post RIDT treatment with oseltamivir.

### Probabilistic Sensitivity Analysis

**Fig 4:**
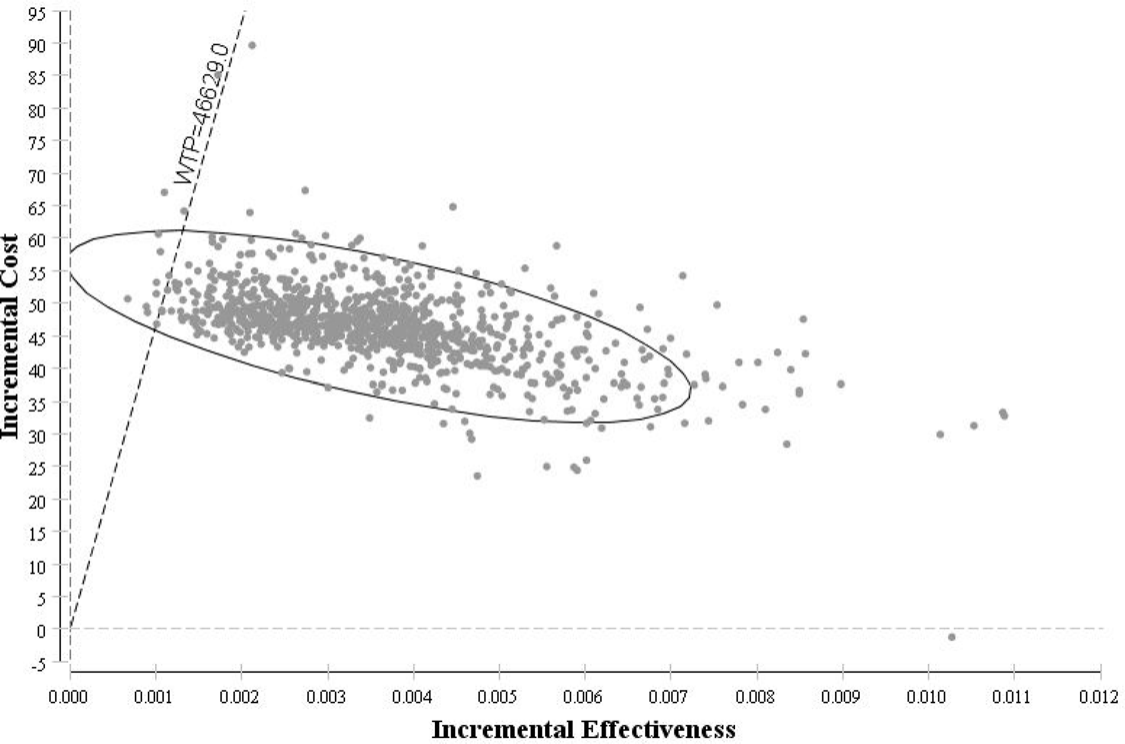
Cost-effectiveness Scatter plot: Empiric treatment with oseltamivir vs. No antiviral therapy.

**Fig 5:**
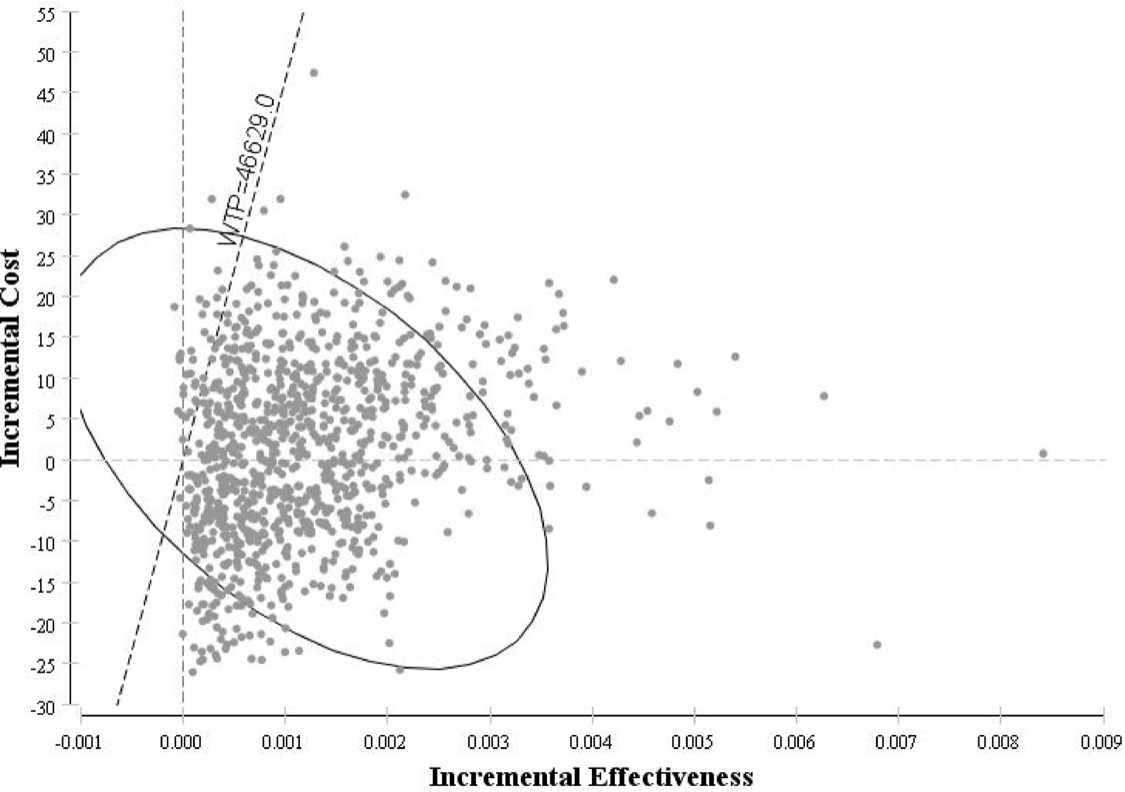
Cost-effectiveness Scatter plot: Empiric treatment with oseltamivir vs. Post RIDT treatment with oseltamivir.

According to the WHO recommendations for cost-effectiveness thresholds, the probabilistic sensitivity analysis shows a 100% probability that empiric oseltamivir treatment would be considered as a very cost-effective strategy compared to the no antiviral therapy. The same was concluded with 99% probability for empiric oseltamivir treatment being a very cost-effective strategy compared to the post RIDT treatment with oseltamivir (Fig 4 and Fig 5).

## Discussion

This analysis has shown that the empiric oseltamivir treatment of children who are suspected to have influenza illness may be a dominant or a very cost-effective treatment strategy in comparison against post RIDT treatment with oseltamivir and no antiviral therapy, respectively in Chinese setting, when seasonal influenza viruses are circulating and antiviral treatment is indicated.

Our analyses were consistent with the cost effectiveness results of oseltamivir treatment for children in the US [5]. However, in this analysis we did not consider the assumptions of oseltamivir resistance and influenza vaccination, as the oseltamivir-resistant influenza were rarely found in children in China [11], and the vaccination rate and willingness of vaccination in children were low in China [12].

Another similar published mathematical modeling based decision analytic cost utility study on healthy and unvaccinated adults aged between 16 and 64 years with an ILI in Canadian setting showed that oseltamivir was both more costly and more effective than supportive care, resulted in a cost effectiveness of $US42,140/QALY. The model was most sensitive to the prevalence of influenza. Decreasing the influenza prevalence from 69% to 50% increased the ICER to $US57,700/QALY, and applying a prevalence of 34% resulted in a further increase to $US68,300/QALY [39].

A similar method was used [40] to evaluate the cost effectiveness of oseltamivir treatment, from the perspective of the government healthcare payer in Canada, in healthy and at-risk adults aged between 18 and 65 years who present to a primary care physician with an ILI within 48 hours of symptom onset. In the base case, diagnostic accuracy was assumed to be 35% when influenza was circulating in the general population, but this was varied from 14% to 68% in sensitivity analyses. Health utilities were assumed to be 1 for healthy individuals, 0.636 for patients with influenza, 0.354 for inpatients with influenza and zero for death. The study showed that the ICER for oseltamivir was highly dependent on the diagnostic accuracy for influenza. Model parameter with the greatest impact on the ICER was the accuracy of diagnosis. The ICER was relatively insensitive to changes in resource utilization and unit cost. This study demonstrated that the sensitivity of the cost effectiveness of oseltamivir to the likelihood of distinguishing influenza from other ILIs in primary care practice, and to the likelihood that patients presenting >48 hours following the onset of symptoms will be prescribed treatment.

A common finding among our analyses and previous publications was the influence of differing assumptions about the prevalence of influenza on the cost effectiveness of influenza management. A similar influence was found with the assumptions around the diagnostic accuracy for influenza based either on clinical symptoms or rapid testing [5, 39, 40]. Assumptions about the disutility of influenza or an ILI also affected the incremental effect.

There are some limitations to our analyses. Firstly we did not consider the oseltamivir-resistant influenza in calculations that might result in a higher ICER for empiric treatment with oseltamivir in comparison against no antiviral therapy, or result in a loss of dominance status in comparison against post RIDT treatment with oseltamivir. During the 2007 to 2008 US influenza season, approximately 11% of circulating seasonal H1N1 viruses tested were resistant to oseltamivir [41]. When oseltamivir-resistant strains are prevalent, the effectiveness of oseltamivir in treating seasonal influenza is limited [5]. However, we did not find any study reported the similar situations in China, relevant studies revealed that the resistance rates of influenza viruses to oseltamivir have been low in different regions of China, and the viruses are sensitive to oseltamivir [42–46]. Furthermore, the recent on-going surveillance data on seasonal influenza virus strains confirmed that the resistance rate to oseltamivir is generally low (1%–3%) globally [47]. Even in the US, no oseltamivir resistance was identified in all circulating influenza viruses through February 2011 of the 2010 to 2011 season [5, 48].

However, we agree that the proportion of circulating oseltamivir-resistant influenza viruses could change and would be impossible to predict [5], we therefore performed a scenario analysis assuming a 29% level of oseltamivir resistance in all circulating influenza viruses, which was consistent with the assumption made in the US study previously conducted [5]. In the results, for a cohort of children not vaccinated against seasonal influenza coming to the physician’s office with ILI, empiric treatment with oseltamivir would cost RMB 25 more than the no antiviral therapy for each child, and produced the greatest number of expected QALYs (0.9952) among all groups. This treatment strategy was estimated to cost additional RMB 9,903 per QALY gained, compared with no antiviral therapy. The empiric treatment with oseltamivir strategy is dominant compared with post RIDT treatment with oseltamivir. The testing strategy (RIDT) was projected to cost RMB45,372 per QALY gained, compared with the no oseltamivir treatment strategy.

This scenario analysis has shown that the empiric oseltamivir treatment of children who are suspected to have influenza illness was still a dominant or a very cost-effective treatment strategy in comparison against post RIDT treatment with oseltamivir and no antiviral therapy, respectively in Chinese setting, when assuming a 29% of oseltamivir resistance in all circulating influenza viruses. The trends of antiviral resistance in influenza, however, need to be kept watching as the on-going monitoring worldwide for this is critical for informing guidance on the antiviral treatment of influenza [5].

Secondly, the model did not consider the influenza vaccination because the vaccination rate and willingness of vaccination in children were low in China [12]. Furthermore, the effectiveness data of the vaccination in children appear inconsistent in China [49]. In a meta-analysis on cohort studies regarding the effectiveness against ILI, the relative risks from the relevant studies varied from 0.17 to 0.56 [49], and there were significant heterogeneities among studies. In another scenario analysis we assumed that up to 50% of children had influenza vaccination and the relative risk of influenza with vaccination was 0.69 [5], the results were still consistent with the above conclusions that the empiric oseltamivir treatment for children with ILI was a dominant or a very cost-effective treatment strategy in comparison against post RIDT treatment with oseltamivir and no antiviral therapy, respectively in the settings of China.

Our analyses have demonstrated the strong association between the cost of medication and the cost effectiveness of the antiviral therapy, the low cost of oseltamivir significantly contributed to the cost-effectiveness of this managing strategy for people with ILI. Other key factors in the effectiveness of antiviral therapy include the prevalence of influenza, sensitivity and specificity of the RIDT, utility of the children with influenza-related complications, and the reduction of the risks of these complications. In summary, based on our analyses, the empiric treatment with oseltamivir strategy is a very cost-effective alternative to the no antiviral therapy, post RIDT treatment with oseltamivir strategy for the children with ILI in local setting of China.

## Supporting Information

S1 Table List of model input parameters and values for base case and sensitivity analyses. (DOCX)
