# Supplementary material for: Oseltamivir Treatment for Children with Influenza-Like Illness in China: A Cost-Effectiveness Analysis
Source: PLoS One. 2016 Apr 15;11(4):e0153664. doi: 10.1371/journal.pone.0153664 (PMC4833415; doi:10.1371/journal.pone.0153664)
Supplement: S1 Table — (DOCX) [file pone.0153664.s001.docx]

| **Variable** | **Description** | **Base case** | **Range** | **Reference** | **Type of distribution** |
| --- | --- | --- | --- | --- | --- |
| pInfluenza | Probability that a child with ILI has influenza | 39.30% | 23.7%-54.90% | [13] | Beta |
| pOtitis | Probability of otitis media for a child with medically attended influenza illness | 18.10% | 11.2%-25.00% | [23] | Beta |
| pPneumonia | Probability of non-hospitalized pneumonia with medically attended influenza illness | 15.12% | 3.3%-28.57% | [24] | Beta |
| pUncomplicated | Probability of other uncomplicated outpatient influenza-related complications | 17.00% | 13.6%-20.40% | [13] | Beta |
| pHospital | Probability of hospitalizations for respiratory condition due to influenza | 1.09% | 0.2%-4.40% | [5] | Beta |
| pLTS | Probability of long-term sequelae after influenza-related hospitalization | 1.00% | 0.3%-3.60% | [5] | Beta |
| pDead | Probability of dying during influenza-related hospitalization | 0.20% | 0.16%-0.24% | [25] | Beta |
| *Probability of medically attended oseltamivir-related adverse events* | | | | | |
| pAEgastro | Gastrointestinal symptoms | 7.45% | 5.96%-8.94% | [26-30] | Beta |
| pAEneuro | Neuropsychiatric symptoms | 0.07% | 0.06%-0.08% | [16] | Beta |
| *Costs (2015 RMB)* | | | | | |
| cOTC | Over-the Countcer medications | RMB21.34 | RMB15.00-RMB100.00 | [31] |  |
| cOseltamivir | Oseltamivir | RMB69.00 | RMB30.00-RMB180.00 | [32] |  |
| cUncomplicated | Outpatient visit for uncomplicated influenza | RMB429.08 | RMB343.26-RMB514.90 | [13] | Log-normal |
| cOtitis | Outpatient visit for otitis media | RMB279.70 | RMB223.76-RMB335.64 | [33, 34] | Log-normal |
| cPneumonia | Outpatient visit for non-hospitalized pneumonia | RMB938.38 | RMB750.71-RMB1,126.06 | [35-39] | Log-normal |
| cHospital | Hospitalization for influenza-related complication | RMB3,934.00 | RMB3,147.20-RMB4,720.80 | [40] | Log-normal |
| cGastro | Outpatient visit for gastrointestinal symptoms | RMB60.00 | RMB48.00-RMB72.00 | [16] | Log-normal |
| cNeuro | Episode cost of neuropsychiatric symptoms | RMB0.00 | RMB0.00-RMB72.00 | [16] |  |
| cTest | RIDT | RMB42.84 | RMB34.27-RMB51.41 | [13] |  |
| cTLS | 1-year cost of long term sequelae | RMB12,622.02 | RMB10,097.62-RMB15,146.42 | [41] | Beta |
| *Characteristics of RIDT* | | | | | |
| TestSens | Sentivity | 26.67% | 21.34%-32.00% | [15] | Beta |
| TestSpec | Specificity | 78.95% | 63.16%-94.74% | [15] | Beta |
| *Oseltamivir treatment efficacy* | | | | | |
| ReducUncom | Reduction in the duration of uncomplicated influenza symptoms | 26.00% | 21%-31.00% | [5] | Beta |
| ReducOtitis | Reduction in risk of otitis media | 40.00% | 32%-48.00% | [4] | Beta |
| ReducPneum | Reduction in risk of pneumonia | 40.00% | 32%-48.00% | [4] | Beta |
| ORdeath | Odds ratio in mortality | 0.82 | 0.58 – 1.17 | [14] |  |
| *Disutility values* | | | | | |
|  | Episode of influenza | 0.005 | 0.002-0.009 | [5] | Beta |
|  | Episode of otitis media | 0.042 | 0.023-0.065 | [5] | Beta |
|  | Episode of pneumonia (or other non-hospitalized complication) | 0.046 | 0.027-0.071 | [5] | Beta |
|  | Hospitalization for pneumonia or other respiratory condition due to influenza | 0.076 | 0.054-0.100 | [5] | Beta |
|  | Gastrointestinal adverse effect | 0.0013 | 0.00065-0.0026 | [5] | Beta |
|  | Neuropsychiatric antiviral-related adverse event | 0.0029 | 0.00-0.10 | [5] | Beta |
| *Utility value* | | | | | |
| uLTS | Utility of long term sequelae | 0.56 | 0.448-0.672 | [42] | Beta |
